# A Re-Examination of *Wolbachia*-Induced Cytoplasmic Incompatibility in California *Drosophila simulans*

**DOI:** 10.1371/journal.pone.0022565

**Published:** 2011-07-25

**Authors:** Lauren B. Carrington, Jeremy R. Lipkowitz, Ary A. Hoffmann, Michael Turelli

**Affiliations:** 1 Department of Genetics and Bio21 Institute, University of Melbourne, Parkville, Victoria, Australia; 2 Department of Evolution and Ecology, University of California Davis, Davis, California, United States of America; University of Poitiers, France

## Abstract

**Background:**

In California *Drosophila simulans*, the maternally inherited Riverside strain *Wolbachia* infection (*w*Ri) provides a paradigm for rapid spread of *Wolbachia* in nature and rapid evolutionary change. *w*Ri induces cytoplasmic incompatibility (CI), where crosses between infected males and uninfected females produce reduced egg-hatch. The three parameters governing *w*Ri infection-frequency dynamics quantify: the fidelity of maternal transmission, the level of cytoplasmic incompatibility, and the relative fecundity of infected females. We last estimated these parameters in nature in 1993. Here we provide new estimates, under both field and laboratory conditions. Five years ago, we found that *w*Ri had apparently evolved over 15 years to enhance the fecundity of infected females; here we examine whether CI intensity has also evolved.

**Methodology/Principal Findings:**

New estimates using wild-caught flies indicate that the three key parameters have remained relatively stable since the early 1990s. As predicted by our three-parameter model using field-estimated parameter values, population infection frequencies remain about 93%. Despite this relative stability, laboratory data based on reciprocal crosses and introgression suggest that *w*Ri may have evolved to produce less intense CI (i.e., higher egg hatch from incompatible crosses). In contrast, we find no evidence that *D. simulans* has evolved to lower the susceptibility of uninfected females to CI.

**Conclusions/Significance:**

Evolution of *w*Ri that reduces CI is consistent with counterintuitive theoretical predictions that within-population selection on CI-causing *Wolbachia* does not act to increase CI. Within taxa, CI is likely to evolve mainly via pleiotropic effects associated with the primary targets of selection on *Wolbachia*, i.e., host fecundity and transmission fidelity. Despite continuous, strong selection, *D. simulans* has not evolved appreciably to suppress CI. Our data demonstrate a lack of standing genetic variation for CI resistance in the host.

## Introduction

*Wolbachia* are maternally inherited intracellular bacteria found in many – and perhaps most – arthropods [1]. The Riverside strain of *Wolbachia* (*w*Ri) was identified in *Drosophila simulans* in the 1980s [2] in southern California, USA. The infection was initially identified by a reduced hatch rate in crosses between males from southern California (Riverside, CA) and females from central California (Watsonville, CA). The reciprocal cross produced significantly more progeny, comparable to the number produced by infected pairs or uninfected pairs. This cytoplasmically inherited incompatibility was associated with *Wolbachia*, first microscopically [3], then via PCR [4]. In laboratory tests, the infection produced no statistically significant fitness consequences other than a reduction in fecundity relative to uninfected flies [5]. Through the late 1980s and early 1990s, *w*Ri spread rapidly into central and northern California [2], [4]–[7]. The *w*Ri infection is now pervasive in California *D. simulans* populations, and its frequency has remained relatively stable after the initial spread [8].

The reduced egg-hatch produced by matings between infected males and uninfected females is termed cytoplasmic incompatibility (CI), and it seems to be the most common reproductive manipulation caused by *Wolbachia* [9], [10]. CI and long-distance dispersal produced the rapid spread of *w*Ri within and among California *D. simulans* populations [2]. A simple population model of CI explained the observed within-population dynamics and equilibrium frequency of *w*Ri in terms of three parameters (see Table 1 of [11]): the maternal transmission rate, 1−*μ*, where *μ* is the fraction of uninfected ova produced by infected females; the relative fecundity of infected females compared to uninfected females, *F*; and the average hatch rate from incompatible fertilizations, *H*, i.e., the relative hatch rate when uninfected ova are fertilized by sperm from infected males. Studies in the early 1990s used repeated experiments with wild-caught flies to estimate these parameters in nature: the average values were *μ*≈0.045, *F*≈1.00, and *H*≈0.55 [4]. While estimates of fecundity in the field generally showed no statistically significant difference between infected and uninfected females, laboratory studies demonstrated a 10–20% fecundity reduction in infected females [5]. Although maternal transmission of *w*Ri was perfect in our laboratory stocks, studies of transmission in the field demonstrated heterogeneity among infected females, with many producing all infected progeny, but many others producing a significant fraction of uninfected progeny [4].

**Table 1:** Infection frequencies from 2008 samples near Davis, California.

Assay	Collection month (2008)	*N*	Infection frequency	95% binomial confidence interval
Maternal Transmission	June/July	146	0.918	(0.861, 0.957)
CI (assay 1)	July	176	0.938	(0.891, 0.968)
CI (assay 2)	August	222	0.937	(0.896, 0.965)
Fecundity	September	538	0.931	(0.906, 0.951)
**Total**		**1082**	**0.932**	**(0.915, 0.946)**

Theory predicts evolution towards a more mutualistic symbiosis, involving changes in one or more of the three key parameters [11]. Selection acts on both the host and *Wolbachia* to foster mutualism. For *Wolbachia* variants that remain mutually compatible, natural selection acts to increase the average number of infected progeny produced by infected females, *F*(1−*μ*) [11], [12]. Under *Wolbachia* evolution, the intensity of cytoplasmic incompatibility, as measured by *H*, might also change, but via pleiotropic effects rather than direct selection [11]–[13]. The effects of natural selection on the host are more complex; but in the absence of pleiotropy, it should act to increase maternal transmission, 1−*μ*; increase female fecundity, *F*; and suppress CI, i.e., increase *H*. The CI phenotype has been described in terms of two bacterial components: the modification of sperm that induces embryo death in incompatible crosses, and the rescue capability of infected eggs, restoring the modification [14]. Both can be influenced by host background effects and *Wolbachia* [10]. Given that both infected and uninfected individuals share the same nuclear genes, a host-mediated evolutionary shift in hatch rate might be the product of changes in males or females. This could involve suppression of CI by the male host genome, or increased resistance to CI by the female genome. We examine both.

Experiments conducted by Weeks et al. [8] indicated that *w*Ri has evolved to increase fecundity. Isofemale lines collected between 2002 and 2004, but maintained in the lab for several generations, showed significant variation in fecundity. Moreover, several showed statistically significant decreases in fecundity when their *Wolbachia* were removed with tetracycline (whereas consistent increases in fecundity with *Wolbachia* removal had been observed in previous studies [5]). One line in which *Wolbachia* increased fecundity, IR2, was chosen for further investigation. Reciprocal introgressions moved *Wolbachia* from Riv88 and IR2 into the alternative nuclear backgrounds. The results demonstrated that IR2 *Wolbachia* significantly raised fecundity on both nuclear backgrounds. Weeks et al. [8] found no evidence for CI between the alternative *Wolbachia*, suggesting that *w*Ri had evolved in the field to become more mutualistic. This interpretation was also supported by the complete sequence identity between the IR2 and Riv88 *Wolbachia* at the rapidly evolving *wsp* locus, which is routinely used for phylogenetic placement of *Wolbachia* strains [15]. CI induction levels across three male ages (5, 10 and 15 days old) in the new flies were not significantly different from those previously reported [4].

In this study, we estimated the current *w*Ri infection frequency in northern California and re-estimated the three key population dynamic parameters: the intensity of cytoplasmic incompatibility (*H*), the fidelity of maternal transmission (*μ*), and the relative fecundity of infected females (*F*). We also asked whether there are differences in CI induction and resistance levels between newly collected flies from the field versus infected and uninfected reference stocks and determined whether observed changes were attributable to the host and/or *Wolbachia*. Our results are discussed in light of theoretical predictions [11].

## Materials and Methods

### Fly stocks and PCR

Four reference *D. simulans* mass-bred laboratory stocks were used: two *w*Ri infected stocks, Riv84 and Riv88, and two naturally uninfected stocks, W88 and Coffs08. Riv84 and Riv88 originated in 1984 and 1988 from Riverside, in southern California, the type location for the *w*Ri infection [2]. W88 originated in 1988 from Watsonville, in central California, our type location for uninfected flies before the *w*Ri infection spread northward [2], [4]. Uninfected Coffs08 originated in 2008 from Coffs Harbour, near the center of the Australian east coast distribution of *D. simulans*.

Collections of *D. simulans* were obtained in 2008 and 2009 from three locations in the Central Valley of California. All three are rural orchards within 10 km of each other, between the towns of Davis and Winters; and all are within 500 m of a riparian corridor, Putah Creek, that separates Yolo and Solano counties. The *w*Ri infection spread northward into this region in the late 1980s [4]. These collections have been named according to their location and year (in subscript). In 2008, we pooled flies from all three orchards and denoted them D_08_; in 2009, we collected only from a Yolo county orchard and denote the sample Y_09_. When referring to a specific line from these collections, the line number is given in place of the collection year, e.g., line 157 collected in Winters in 2008 is D157. A letter following the line number indicates *Wolbachia* transmission characteristics, ‘P’ indicates partial transmission (i.e., some uninfected isofemale sublines were obtained), and ‘U’ indicates an uninfected line. For all field assays, flies were crossed to reference stocks or placed individually in vials to create isofemale lines within hours of field collection.

### Infection status via PCR

To screen for *Wolbachia*, we used a rapid method of DNA isolation for PCR amplification. A 120 µl aliquot of buffer (10 mM TrisHCl, 1 mM EDTA, 25 mM NaCl, with 0.005% Proteinase K) was used for grinding individual flies for two minutes in 1.1 ml deep-well plates (Axygen, USA) using 3 mm glass beads in either a Mixer Mill (Retsch, USA) or Genogrinder (OPS Diagnostics, USA), depending on the lab in which extractions were done. Samples were incubated for 30 minutes to an hour at 37°C followed by 4 minutes at 95°C, before being immediately placed on ice. After two minutes of centrifugation at 13,000 rpm, the supernatant was suitable for standard PCR.

The infection frequency from the field collections was determined by the PCR protocol from Zhou et al. [15] using *wsp* primers. Primers amplifying the single-copy *Drosophila* nuclear gene suppressor of sable (*su(s)*) [16] were used as a positive control [4]. The infection frequency was estimated using all of our summer 2008 field collections.

The *wsp* gene from infected isofemale lines established in 2008 was sequenced to confirm that the infecting *Wolbachia* strain was *w*Ri. Extracted DNA was amplified for the *wsp* gene and sent to Macrogen, Korea, for purification and sequencing. All of the infected D_08_ strains used in the CI assays (including backcrosses) contained *w*Ri.

### CI assays using field-collected males

To quantify CI intensity, field-collected males were brought into the laboratory and mated to reference laboratory stocks within hours of collection. Over two independent assays (initiated 23 July 2008 and 5 August 2008), 500 wild-caught males were tested. Each assay began with 250 field-collected males. The four sets of crosses undertaken in both assays were identical: (1) 200 field males mated to W88 females on the first day, then mated to R88 females on the second day; (2) 50 field males mated to W88 females on two successive days to test the effects of remating on CI [17], [18]; (3) 20 W88 males mated to W88 females on the first day and Riv88 females on the second day to determine whether egg hatch from compatible crosses depended significantly on the origin of the uninfected male (and to look for inbreeding effects); and (4) a second group of 10 W88 males mated to W88 females on two successive days (to further check for inbreeding effects). After 24 hours with the second female, males were removed and frozen at −80°C for later testing of infection status.

Females were aged between 3–5 days after eclosion before mating. Females were allowed to lay eggs on spoons for 48 hours, with fresh spoons provided after 24 hours. The spoons contained molasses medium with a live yeast suspension to encourage egg-laying. After each 24-hour laying period, all eggs laid on the spoons were left 24 hours for hatching before the spoons were frozen. The number of eggs laid by each female was counted, along with the number of hatched eggs (or larvae). Hatch rates were compared between incompatible and compatible crosses and among classes of compatible controls. We excluded from the analyses egg data from females that laid fewer than 10 eggs over two days and data from wild-caught males whose infection status could not be confirmed.

### CI assays comparing newly collected stocks to Riv88 and W88

We performed three sets of assays to determine whether CI levels had evolved.

#### Assay 1: CI induction and susceptibility using D_08_ isofemales

Field-collected females were used in 2008 to assess maternal transmission of *Wolbachia*. Some of the resulting infected and uninfected isofemale lines were subsequently assayed for CI to determine: (1) whether infected males from D_08_ stocks produced CI levels that differed from our reference Riv88 stock, and (2) whether females from uninfected D_08_ stocks differed from our reference W88 stock in their susceptibility to CI.

CI levels induced by lab-reared D_08_ males were compared to those induced by our laboratory stock, Riv88. Five D_08_ isofemale lines, assayed in this study for maternal transmission of *Wolbachia*, were tested. Three of the five showed “perfect” transmission (i.e., produced no uninfected sublines) (D157, D331 and D352), and two showed “partial” (<100%) transmission (D29P and D287P). The ability of five uninfected D_08_ isofemale lines (D350U, D362U, D365U, D372U and D373U) to resist CI was compared to that of W88 females.

Our experimental design for CI induction involved mating males from Riv88 and each of the five D_08_ infected lines to W88 females. We also mated males from each of the infected D_08_ lines to females from one of the five uninfected D_08_ lines. Conversely, females from W88 and each of the D_08_ uninfected lines were mated to Riv88 males to test for variation in susceptibility to CI. Crosses within each of the five infected and uninfected D_08_ lines were included as controls.

For these CI assays, flies were reared at a controlled density prior to testing. Groups of 25 eggs were collected from treacle-based media with a yeast suspension and transferred to fresh vials containing 10 ml of cornmeal medium. CI was tested in virgin males aged 7 and 14 days. Females were between three and five days of age. We performed 20 replicates of each incompatible cross, and 10 replicates of each compatible cross. The D_08_ flies had been reared in the lab for a maximum of four generations. As in the field CI assays, females were allowed to deposit eggs on spoons for 48 hours (spoons changed after 24 hours), and hatch for a further 24 hours, before being frozen and later counted to determine hatch rate.

#### Assay 2: CI induction using Y_09_ isofemales

We also assessed variation in male-associated CI induction using our Y_09_ lines. A total of 100 isofemale lines, established from wild-caught females, were tested. The F1 males were collected as virgins from each line and aged seven days. One generation later, we retested the level of CI produced by 14 of these lines, 7 that initially showed the highest levels of CI and 7 that initially showed the lowest levels of CI. The experimental design followed our lab CI assays, with between 5 and 10 replicate crosses per line.

#### Assay 3: retest CI induction of Y_09_ isofemales in Melbourne

The 14 lines initially identified as high versus low CI in Assay 2 were shipped to Melbourne for further testing. One of the seven initial low-CI lines was lost. A subset of the Y_09_ isofemale lines was retested with increased replication in Melbourne to assess whether the differences observed in Davis were repeatable and attributable to host versus *Wolbachia* effects. Again, these males were placed with virgin females of the W88 line. The flies were tested about ten generations after being removed from the field. As elaborated below, three lines from the initial “high” CI group and three lines from the initial “low” CI group were used in reciprocal crosses with Riv88 to test for maternally inherited variation in CI induction, while controlling for host nuclear genetic effects.

### Host versus *Wolbachia* contributions to CI variation

We performed two assays to determine whether variation among males from D_08_ and Y_09_ lines in CI induction and differences from our old Riverside stocks were most likely attributable to *Wolbachia* (cytoplasmic) or *Drosophila* (nuclear) effects. Each assay involved introgression or reciprocal crosses. As discussed below, these designs do not directly distinguish *Wolbachia* effects from other maternally inherited factors.

#### Introgression assays between Riv88 and D157

Our assay of male-induced CI variation in D_08_ resulted in the identification of line D157, which produced only infected sublines, yet induced less intense CI than Riv88. Reciprocal backcrossing was used to place the *Wolbachia* from Riv88 versus D157 onto one another's nuclear genetic background. The reciprocal replacements were denoted D157^Riv88^ and Riv88^D157^, where the superscript denotes the *Wolbachia* source. Four generations of backcross resulted in a predicted replacement of over 93% of the host nuclear background. The backcross lines and the parentals (i.e., Riv88^Riv88^ and D157^D157^) were assayed together.

CI assays used seven-day-old males. As with the other lab-based CI assays, single virgin males and females were placed together in a vial to mate, and the female was allowed to deposit eggs for 24 hours. The females were three to four days old. For each line, males were crossed to both W88 and Riv88 females. There were eight to ten replicates per cross. A second assay using these same lines was performed three generations after the first, with 16 to 30 replicates for each incompatible cross.

#### Reciprocal cross assays using Y_09_ lines and Riv84

We assayed CI with F1s from reciprocal crosses between six of the initially screened Y_09_ lines and Riv84. There were seven to eight replicates of each cross type, and only the replicates that produced 10 or more eggs were included in the analyses. F1 males were mated to uninfected Coffs08 females to produce incompatible crosses.

### Maternal Transmission

Maternal transmission of *Wolbachia* was assayed twice using wild-caught D_08_ females, following an improved version of the Turelli and Hoffmann [4] protocols. More than 100 *D. simulans* females were collected over one month, from mid-June to mid-July 2008. The frequency of uninfected individuals produced by infected females was determined by establishing multiple F1 sublines from the wild-caught females, then scoring the F2, as described below. Each field-collected female was allowed to lay eggs for around six days in the lab prior to mating her to an uninfected reference male (W88), and then allowing her to lay eggs for another six days. By remating field-collected females to W88 males, we retested the hypothesis that uninfected ova produced by an infected mother show the same susceptibility to CI as do ova from uninfected mothers [4]. This susceptibility must be considered to accurately assess *μ* because the high *w*Ri infection frequency in California means that most fertilizations involve sperm from infected males. If an infected female produces uninfected ova, these ova may be subject to CI and not hatch. This would lead to underestimation of *μ* from the fraction of uninfected sublines. Mating wild-caught females to uninfected males should produce more accurate estimates.

Each field female's F1 progeny were aged and allowed to mate with each other, both before and after the field-caught female was mated to the W88 male, and up to ten already mated F1 females were placed individually in fresh vials to establish F2 sublines. Initially, one F2 female from each subline was tested for infection status. A subline was scored as infected if the individual tested positive for *Wolbachia*. Sublines were scored as uninfected only after four additional females from the same subline were found to be uninfected. The percentage of infected F2 progeny was estimated for each field female based on the fraction of uninfected versus infected sublines produced before and after mating with a W88 male.

After the initial maternal transmission experiment, ten D_08_ lines, five infected and five uninfected, were selected for continued maintenance and used in the CI comparisons discussed previously under “Assay 1: CI induction and susceptibility using D_08_ isofemales”. These five infected lines consisted of three *Wolbachia*-infected lines that produced 100% infected sublines and two that produced <100% infected sublines.

### Fecundity in the field

Relative fecundity of infected and uninfected wild-caught females was estimated using flies collected in August 2008. A total of 538 *D. simulans* females from the field was obtained and assayed for fecundity over four days. Single females were placed in vials containing spoons, replaced daily. Spoons were frozen until the eggs could be counted. The F1 progeny of these lines were used to check the infection status of the field females. We discarded data from females who died during the four-day egg-laying period.

Body size and fecundity are typically positively correlated in *Drosophila* (e.g. [19]). Thorax measurements were made for 441 field females, as an index of body size. The thorax of each female was photographed and measured using the program AutoMontage, after storage in 70% ethanol. Data on thorax length and fecundity for all field females were used to look for associations between them and for effects of *Wolbachia* on size or fecundity.

### Infection frequency

We estimated the incidence of *Wolbachia* in the field in northern California in 2008. We tested 398 field-collected males from the field CI assay and 538 field-collected females from the fecundity assay. We also assessed the infection status of 146 field females collected for our maternal transmission study by examining their F1 male progeny. In this case, when a negative PCR result was initially obtained for an isofemale line, four more males were tested.

### Statistical analyses

For all CI assays, hatch rates were computed only when females produced at least 10 eggs. For the initial assays involving field males and comparisons to laboratory males, data were often not normally distributed, so nonparametric tests were used in all initial surveys of lines. In experiments where the same males were mated to different females on successive days, we undertook pairwise comparisons of percentage hatch rates using Wilcoxon paired tests to test for differences in CI levels in assays on day 1 versus day 2. For the remaining comparisons of CI, we considered different crosses and these were compared with nonparametric Mann-Whitney tests (for two-group comparisons) or Kruskal-Wallis tests (for more than two groups). For estimates of CI intensity (*H*) and failure of maternal transmission (*μ*), bias-corrected and accelerated (BC_a_) bootstrap confidence intervals were obtained based on 10,000 bootstrap replicates (see [4] and Ch. 14 of [20]).

In CI assays following crosses between different strains to determine the effects of *Wolbachia* or nuclear background, hatch rate data were normally distributed; and we used parametric tests to compare host and *Wolbachia* background effects. ANOVAs were used to separate these effects.

To compare infection frequencies across collections, we used contingency tables and computed the chi-square statistic. Exact confidence intervals based on the binomial distribution were computed for the resulting frequency estimates [4].

## Results

### Cytoplasmic incompatibility (CI) assays with field-collected males

#### Field assay 1

As noted above, there were three classes of males: (1) 200 field males mated to W88 females on the first day, then mated to R88 females on the second day; (2) 50 field males mated to W88 females on two successive days; (3) 20 W88 males mated to W88 females on the first day and Riv88 females on the second day. We included only individual crosses that produced at least 10 eggs. The first two groups of males were used to estimate levels of CI, controlling for male fertility and examining the effects of male age and/or remating, while the last cross tested the fitness of the W88 line, looking for possible inbreeding effects. We also included crosses between W88 males and W88 females on both days, but hatch rates from these crosses did not differ significantly in either assay and are not presented.

As expected for the first class of males, matings on the first day, between infected males and uninfected females, produced a lower hatch rate (38%, *N* = 41) than matings on the second day between the same infected males and infected females (75%). Considering only males who produced at least 10 offspring from each cross, the difference is significant in a paired Wilcoxon test on hatch rates (*Z* = 4.20, *P*<0.001). In contrast, for uninfected field males, there was no significant difference (*Z* = 0.674, *P* = 0.50) between hatch rates from day 1 matings with uninfected females (65%) and day 2 matings with infected females (75%), although there were only five data points for comparison. When all crosses are compared by a Kruskal-Wallis test including those that produced sufficient offspring on either day 1 or day 2, there was no difference in CI between the three sets of compatible crosses involving field males (*X^2^* = 0.37, df = 2, *P* = 0.831); these consisted of uninfected males mated to uninfected (hatch rate 71%, *N* = 6) or infected females (73%, *N* = 9) and infected males mated to infected females (80%, *N* = 131). We combined all three classes of compatible crosses involving field males to estimating field CI levels. However, for the incompatible crosses, we considered only those from day 1, because CI levels decline with age [4] and remating [17], and a difference between days was detected in assay 2 in paired crosses (below).

Using our pooled compatible crosses, we obtain a point estimate of *H*, the relative hatch rate from incompatible crosses, of 0.42, with a bias corrected and accelerated (BC_a_, [20], Ch. 14) 95% bootstrap confidence of (0.33, 0.53).

In the third set of crosses, a Mann-Whitney U test indicated an unexpected statistically significant difference (*Z* = 2.098, *P* = 0.036) between hatch rates for W88 males mated to W88 females on days 1 (93%, *N* = 12) and Riv88 females on day 2 (83%, *N* = 15). We assign no biological significance to this anomalous result, which was not repeatable in our second field assay.

#### Field assay 2

For the first set of males, infected males produced a lower hatch rate (*Z* = 9.74, *P*<0.001) when mated to uninfected females on day 1 (26%, *N* = 130) than when mated to infected females on day 2 (86%), as expected with CI. For the uninfected field males, there was no difference (*Z* = 1.57, *P* = 0.116) between days 1 (75%, *N* = 7) and 2 (86%), as expected for compatible crosses. For the second set of infected field males mated to uninfected females on days 1 and 2, there was a significant difference by paired Wilcoxon test (*Z* = 3.79, *P*<0.001) between days due to a lower hatch rate on day 1 (37%, *N* = 40) compared to day 2 (49%). This increase may be attributable to either male age or remating. We also had five uninfected males in the second set of crosses that produced offspring on both days, and these did not differ significantly between days (*Z* = 0.67, *P* = 0.50).

As for assay 1, we combined all three classes of compatible crosses in estimating field CI rates. However, there was a significant difference among the classes (*X^2^* = 29.83, df = 3, *P*<0.001), because there was a lower hatch rate in crosses between uninfected field males and uninfected females on day 1 (66%, *N* = 12) and day 2 (59%, *N* = 5) compared to crosses on day 2 involving infected field males and infected females (84%, *N* = 156) and uninfected field males and infected females (87%, *N* = 9). The reason for these differences is unclear unless some field males scored as uninfected carried a low level *Wolbachia* infection that was not detected.

For the compatible cross, we considered only males that produced at least 10 offspring on day 1. These led to a point estimate for the intensity of CI in the field of *H* = 0.35 and a 95% BC_a_ bootstrap confidence interval of (0.30, 0.40).

For the third set of crosses with W88 males, the Mann-Whitney U test was not significant (*Z* = 0.260, *P* = 0.795), with a high hatch rate after crosses to both W88 females on day 1 (81%, *N* = 14) and Riv88 females on day 2 (80%, *N* = 14).

### CI assays comparing newly collected stocks to Riv88 and W88

We performed three sets of assays; the first assay addressed two separate issues, discussed in succession below.

#### Assay 1a: CI induction by infected males, D_08_ lines versus Riv88

**Figure 1:**
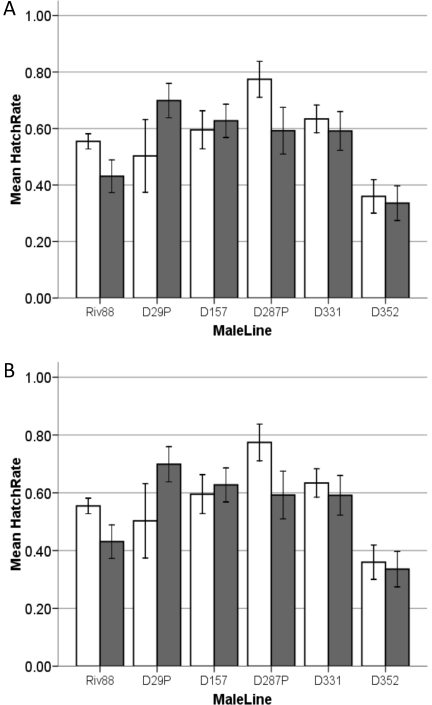
CI induction of old (Riv88) and new (D_08_) males on new (D_08_) and old (W88) uninfected females. Females of newly collected lines are shown in white bars, females from W88 are shown in grey. Error bars show ±1 SE of the mean. Bars indicate the mean hatch of uninfected females when mated to infected males who are: **A**) seven days old, and **B**) fourteen days old.

We compared levels of CI induced at ages 7 and 14 days by Riv88 males versus males from five infected D_08_ lines. The D_08_ lines were selected based on their maternal transmission characteristics (see section on Maternal Transmission below). We used three lines showing perfect transmission and two showing partial transmission, as assessed using isofemale lines established directly from the field. Figure 1 presents the data from all of our incompatible crosses, distinguishing those that involved W88 females (grey bars) versus uninfected D_08_ females (white bars). None of the adjacent grey and white bars (reflecting a specific infected male line mated to two different uninfected female lines) differ significantly (statistics not shown). Hence, our comparisons below pool the results involving W88 and uninfected D_08_ females. This lack of variation in uninfected female susceptibility to CI is elaborated below.

For day 7, as expected, incompatible crosses produced consistently lower hatch rates (average 29%) than compatible crosses (73%) (Mann-Whitney: *Z* = −11.308, *P*<0.001). More interestingly, Figure 1A shows that Riv88 males had a lower average hatch rate, 16.9% (*N* = 102), than males from the five new infected lines, which averaged 37.8% (*N* = 178). There was a significant effect of male line (Kruskal-Wallis: *X^2^* = 72.27, df = 5, *P*<0.001) and significant heterogeneity among the five infected D_08_ lines (Kruskal-Wallis: *X^2^* = 47.05, df = 4, *P*<0.001).

Two of the five infected D_08_ lines assayed, D29P and D287P, showed incomplete maternal transmission. Assuming the males inherited relatively few or no *Wolbachia* (see [4]), we expected them to induce less intense CI than did the lines with perfect transmission. As expected, these two lines produced the highest hatch rates of the five D_08_ lines tested (Figure 1A). The perfectly transmitting and partially transmitting D_08_ lines are different from each other. Overall, the perfect lines have higher CI than the partial lines (*Z* = 6.028, *P*<0.001). However, even the perfectly transmitting lines produced higher hatch rates than Riv88. Because D157 produced the lowest level of CI among the perfect transmission lines, it was chosen for further study. At age 7, D157 had a mean hatch of 0.333, with a 95% bootstrap confidence interval of (0.276, 0.403), versus 0.188 (0.150, 0.224) for Riv88.

At day 14, CI induced by infected males declined significantly, as expected [2]. The hatch rate of the incompatible crosses increased (mean 0.586, 95% confidence interval (0.556, 0.615)), but remained significantly lower than that observed for compatible crosses (0.721, (0.665, 0.770)) (Mann-Whitney: *Z* = −5.59, *P*<0.001). Male lines still differ in their CI intensities (Kruskal-Wallis: X^2^ = 39.62, df = 5, *P*<0.001; Figure 1B), but the males from the two lines with imperfect transmission no longer differ significantly from Riv88.

#### Assay 1b: CI susceptibility of uninfected females, D_08_ lines versus W88

**Figure 2:**
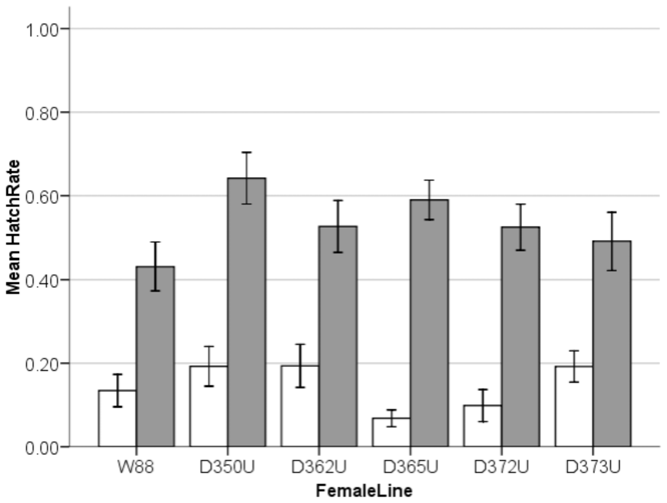
Susceptibility to CI of uninfected females from recently collected D_08_ lines and the old lab-maintained W88, to CI. Mean hatch of females from seven-day-old (white bars), and fourteen-day-old (grey bars) infected males. Error bars show ±1 SE of the mean.

The susceptibility to CI of recently collected, uninfected isofemale lines was assessed relative to W88 females. Figure 2 compares the levels of CI induced by Riv88 males, aged 7 and 14 days, on W88 and all five uninfected D_08_ lines used in Figure 1 (elaborating the comparison shown in the Riv88 columns of Figure 1). As expected from Figure 1, Figure 2 shows that the female line did not influence the hatch rate in incompatible crosses at age 7 (Kruskal-Wallis: *X^2^* = 5.55, df = 5, *P* = 0.352) or 14 (*X^2^* = 4.92, df = 5, *P* = 0.425). Thus, Figure 2 indicates that females from the new uninfected isofemale lines did not differ from W88 in their susceptibility to CI induced by Riv88 males; whereas Figure 1 shows that new (D_08_) and old (W88) uninfected lines are equally susceptible to newly collected infected males (D_08_).

#### Assay 2: CI induction by males from Riv88 versus infected Y_09_ isofemales

We repeated CI assays in 2009 using newly established Y_09_ isofemale lines. Virgin F1 males from 100 Y_09_ lines were screened for CI induction at age seven days. After our initial screening, we maintained 14 isofemale lines for further testing: 7 that indicated the highest level of CI (with mean egg hatch ranging from 0.043 to 0.155, overall mean 0.091) and 7 that indicated the lowest level of CI (mean egg hatch ranging from 0.28 to 0.585, overall mean 0.433). These 14 lines were tested again for seven-day-old CI induction the next generation. The average hatch rates for the seven “low” lines now ranged from 0.102 to 0.354, with an overall mean of 0.254; whereas the seven “high” lines now ranged from 0.232 to 0.637, with an overall mean of 0.344. These two groups differed significantly (one-sided Wilcoxon, *P* = 0.014). However, there was considerable variation in the point estimates of CI intensity across the two replicates. If we rank lines as “high” or “low” based on whether the mean hatch rates are in the bottom six or top six, only lines Y6, Y30 and Y41 were consistently high across both tests; and only Y47 and Y83 were consistently low across both tests.

#### Assay 3: retest CI induction of Y_09_ isofemales in Melbourne

**Figure 3:**
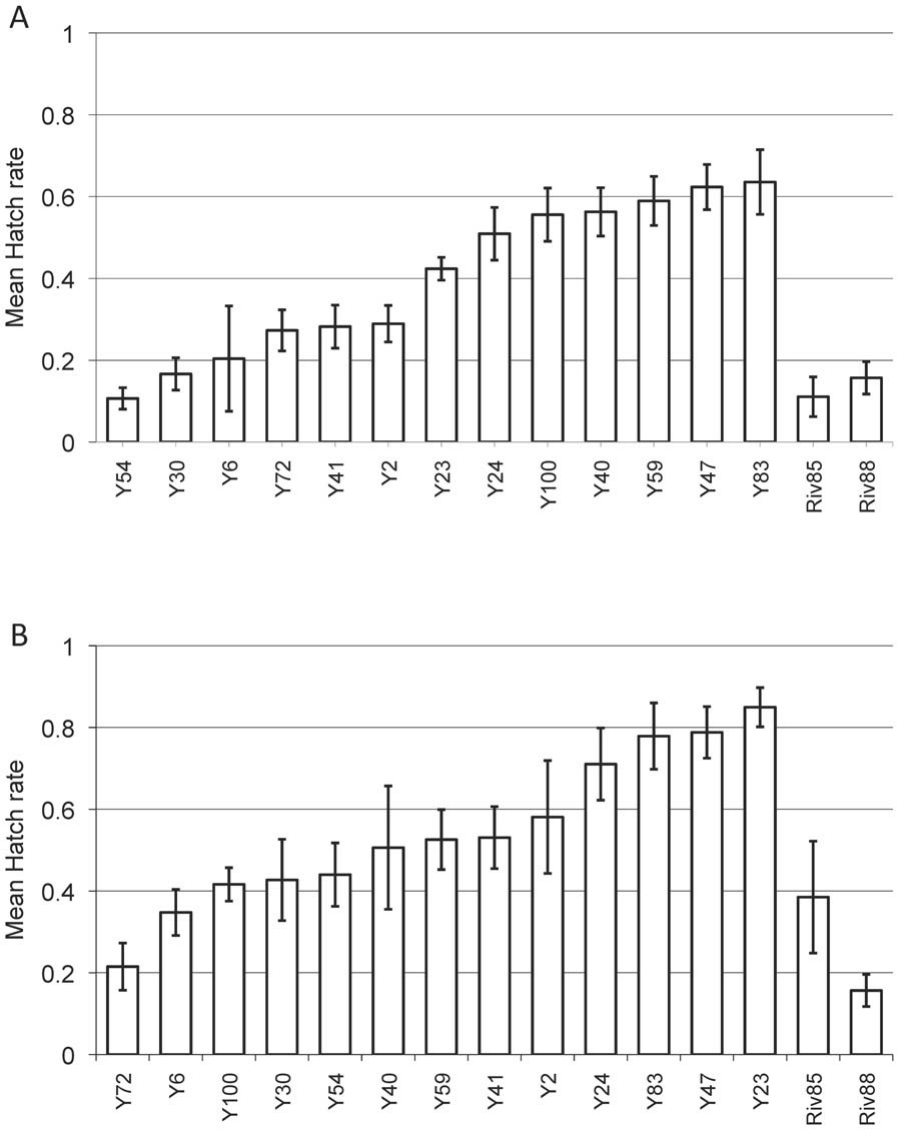
Hatch rates of 13 Y_09_ lines tested in the lab, demonstrating variation among these lines and their differences from Riv85 and Riv88. Males are aged **A**) 7 days; and **B**) 14 days post-eclosion.

One of the seven initial “low-CI” Y_09_ lines was lost. We retested the remaining 13 lines with greater replication (eight replicates per cross with uninfected females) alongside Riv84 and Riv88. All lines were confirmed as infected. The level of CI they produced tended to be lower than that produced by Riv84 or Riv88 (Figure 3). Differences between the two old Riv lines and the new Y_09_ lines were significant on the basis of a one-tailed test for both day 7 (*Z* = 1.87, *P* = 0.031) and day 14 (*Z* = 1.87, *P* = 0.031).

### Dissection of variation in male CI-induction

#### Introgression assays between Riv88 and D157

**Figure 4:**
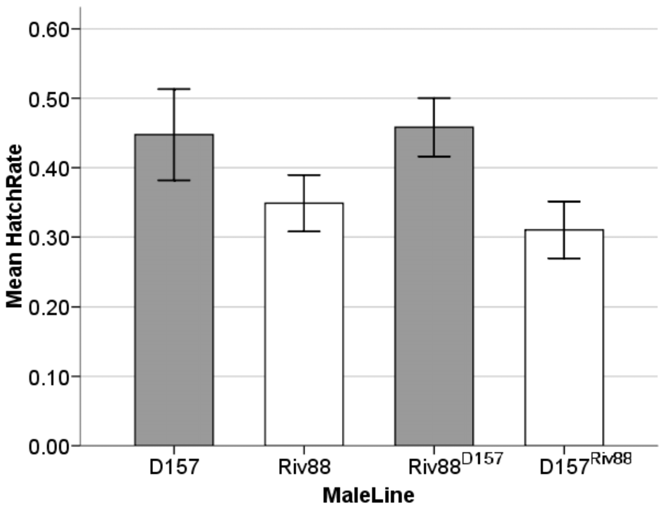
Mean levels of CI induction for males aged 7 days, contrasting the parental strains D157 and Riv88, and the reciprocal backcrosses created from these lines. Host background (normal script) and the infecting *Wolbachia* (superscript) for each line are noted. The grey and white highlight lines that carry *Wolbachia* from the same source. Error bars show ±1 SE of the mean.

This assay addressed whether differences in the host, the *Wolbachia*, or both genomes produced the lower levels of CI for D157 in comparison to the Riv88 (Figure 4). An ANOVA showed a significant effect of *Wolbachia* (and/or other cytoplasmically inherited factors) (*F*_1, 97_ = 6.874, *P* = 0.010). The mean hatch rate for males carrying Riv88 *Wolbachia*, irrespective of their host background, was 0.327 (95% bootstrap confidence interval (0.273, 0.386)), while males carrying *Wolbachia* from the D157 line showed a mean of 0.455 (0.387, 0.523). No effect of host genetic background (*F*_1, 97_ = 0.274, *P* = 0.602) was found. The Riv88 background had a mean hatch rate of 0.409 (0.350, 0.470), and the D157 background had a mean hatch of 0.356 (0.289, 0.428).

#### Reciprocal-cross assays using Y_09_ lines and Riv84

**Figure 5:**
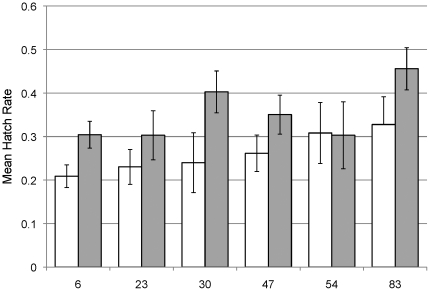
Hatch rates produced by seven-day-old F1 males from reciprocal crosses between Riv84 and the infected Y_09_ lines. White bars show crosses carrying “old” *Wolbachia* (Riv84 females mated with males from the line indicated), while grey bars are crosses carrying “new” *Wolbachia* (females from the lines indicated crossed with males from Riv84).

We further explored the effects of maternally inherited factors while controlling for host nuclear background by setting up a single generation of reciprocal crosses between six Y_09_ lines and Riv84. When all the new lines were considered together and compared to Riv84, we found a significant difference between the reciprocal crosses (*F*_1,100_ = 9.404, *P* = 0.003), with lower hatch rates associated with Riv84 matrilines. For five of the six lines, means indicate higher hatch rates when the female parent was from a Y_09_ line versus Riv84; while for the remaining line (Y54), means were essentially identical between reciprocal crosses (Figure 5). When reciprocal crosses were compared with one-tailed *t* tests, there were significant differences between crosses involving Y6 (*t* = 2.37, df = 17, *P* = 0.015) and Y30 (*t* = 1.95, df = 15, *P* = 0.035). The Y6 strain had previously shown consistently low CI levels compared to the old Riv strains (see above). There were marginally non-significant differences for crosses with Y47 (*t* = 1.46, df = 17, *P* = 0.081) and Y83 (*t* = 1.59, df = 12, *P* = 0.079), while the differences were not significant for Y23 (*t* = 1.067, df = 14, *P* = 0.150) or Y54 (*t* = 0.76, df = 15, *P* = 0.225).

Three of these lines were identified in our initial screen of CI-induction assay 2 as producing high levels of CI (Y6, Y30 and Y54) and three were initially identified as producing low levels of CI (Y23, Y47, Y83). However, when compared in this reciprocal-cross assay, the two groups did not differ significantly. Only Y6 showed consistently high CI across both tests in CI-induction assay 2 and CI-induction assay 3 (Figure 3); whereas both Y47 and Y83 were consistently in the lower CI groups in all three tests. Overall, our data indicate a high level of variation in CI intensities across replicate assays.

### Maternal transmission

From 477 wild-caught females, 146 isofemale *D. simulans* lines were established. The sample included uninfected females, infected females that produced only infected progeny (“perfect” transmission) and those that produced both infected and uninfected progeny (“imperfect” transmission). Maternal transmission was initially assessed using F1 progeny produced by females directly from nature. Maternal transmission was reassessed after the field females were aged and mated to W88, our reference uninfected stock. Only isofemale lines that produced eight or more F2 sublines were used in our analyses.

Using F1 produced from field matings, 77 infected females produced a total of 712 F2 sublines, of which 11 were uninfected. Because most of these field females will have mated with infected males, many of their uninfected ova would be subject to CI. Let *ν* denote the fraction of uninfected F2 sublines produced by wild-mated, infected females. Our data produce the estimate *ν* = 0.015, a lower bound for *μ*. Only 3 of the 77 wild-caught, infected females (3.8% of total) produced the 11 uninfected sublines. A more careful estimate involves weighting the data from each female by the number of F2 sublines she produced. This also produces *ν* = 0.015. Bootstrapping the data from the collections of sublines produced by individual wild-caught females produces a 95% BC_a_ bootstrap confidence interval of (0.003, 0.070). Note that this confidence interval is much broader than the 95% binomial confidence interval, (0.008, 0.027), based on 11 uninfected F2 sublines out of 712. The much larger BC_a_ confidence interval more appropriately accounts for sampling variance because of the extraordinary heterogeneity of maternal transmission among females. Out of 77 wild-caught, infected females that produced at least 8 F2 sublines, 74 females produced only infected F2 sublines. In contrast, of the remaining three females, one produced only two infected sublines out of ten, the other two produced 8/10 and 8/9 infected sublines.

**Figure 6:**
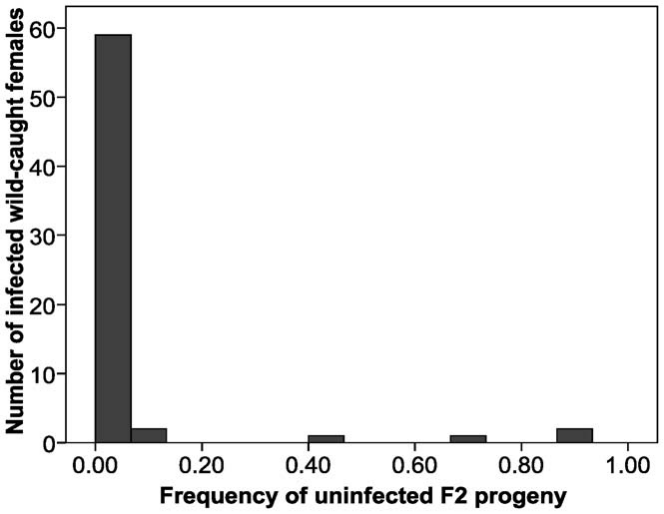
Histogram showing the number of infected females from nature that yielded different frequencies of uninfected F2 progeny after mating to W88 males in the lab.

If uninfected ova from infected females are subject to CI, *v* underestimates *μ*, the fraction of uninfected ova produced by infected mothers. To estimate *μ* more accurately, the field-caught females were aged and remated to W88 males in the lab, then F2 sublines were established from the resulting F1. Using F1 produced from W88 matings, 65 infected females produced a total of 588 F2 sublines, of which 29 were uninfected. Only 6 of the 65 W88-remated, infected females (9.2%) produced the 29 uninfected sublines. These data produce a weighted-average estimate of *μ* = 0.048 (unweighted, 0.049) and a 95% BC_a_ bootstrap confidence interval of (0.016, 0.116), based on 10,000 bootstrap replicates (over the groups of sublines produced by individual field-caught females). Again, the bootstrap confidence interval is much larger than the corresponding 95% binomial confidence interval of (0.033, 0.070), because of the degree of heterogeneity in transmission fidelity across W88-remated females. Of the six females that produced both infected and uninfected F2 sublines, two produced only one infected subline out of nine, a third produced three infected out of ten, a fourth produced five infected out of nine, and the remaining two produced 7/8 and 9/10 infected. Hence 4 of 65 wild-caught, infected females accounted for most of the imperfect maternal transmission. The F2 data from infected females remated to W88 males are illustrated in Figure 6.

The most comparable data from Turelli and Hoffmann [4] are from their fourth maternal transmission experiment, reported in their Figure 4. It shows that 15 out of 59 females from nature, remated to uninfected males, produced both infected and uninfected progeny. Those data involved eliminating one female who produced only 1 infected subline out of 11, so overall 16 of 60 females collected in 1993 produced infected and uninfected sublines. In contrast, only 6 of 65 females in 2008 produced both infected and uninfected F2. Using a χ^2^ test, this difference is statistically significant (*P*<0.05). However, as elaborated in our Discussion, much greater differences were observed between two of the other maternal transmission studies reported in Turelli and Hoffmann [4]. Despite significant heterogeneity among estimates, our new point estimate, *μ* = 0.048, is similar to the composite estimate, *μ* = 0.042, produced by Turelli and Hoffmann [4] from four separate experiments.

Although the binomial confidence intervals for our new estimates of *ν* and *μ*, from wild-mated versus W88-remated females respectively, do not overlap, the more valid BC_a_ confidence intervals do. Hence, even though the corresponding point estimates, 0.015 versus 0.048, are appreciably different, they are not statistically significantly so. (This assertion is supported by a permutation-based test, analogous to that in [21], which asks whether the observed difference in the estimates is consistent with a null distribution of differences obtained by randomly partitioning the subline data pooled from both experiments.) Nevertheless, as discussed below, our data indicate that uninfected ova from infected females are subject to CI, as demonstrated by Figure 4 of [4].

### Fecundity

The field assay for fecundity assessed relative egg numbers produced by infected and uninfected females from nature. The fecundity of 538 *D. simulans* females was tested, over four days. This sample contained 93.12% infected females. During the assay, 35 infected females died, leaving 466 infected and 37 uninfected females. The fecundity of infected and uninfected females did not differ significantly (*F*_1, 501_ = 0.96, *P* = 0.75). Infected females laid on average 13.56 (±0.27 SE) eggs per day, while uninfected females laid 13.87 (±1.02) eggs, producing a point estimate for *F*, the relative fecundity of infected females, of 0.98, and a 95% BC_a_ bootstrap confidence interval of (0.85, 1.15), based on 10,000 bootstrap replicates.

Thorax length was measured for 441 females. This was significantly correlated with fecundity (*r^2^* = 0.294, *P*<0.001). When thorax length was included as a covariate, it significantly influenced egg production (*F*_1, 438_ = 41.71, *P*<0.001), as expected; but there was no significant difference in the mean thorax lengths of infected and uninfected females (*F*_1, 439_ = 0.786, *P* = 0.376).

### Infection Frequency

For our 2008 collections, the infection frequencies were homogeneous across assays (χ^2^ test, 3 df, *P*>0.8). In a sample of over 1000 (Table 1), we found 93.2% infected with *w*Ri, with a binomial confidence interval of (0.915, 0.946). These data are obviously consistent with the overall infection frequency of 0.93 reported by Weeks et al. [8] from 654 individuals sampled in 2004 from four populations throughout California, including one near Davis and two from southern California.

## Discussion

### Cytoplasmic Incompatibility

#### Field estimates

Our new estimates of hatch rates from incompatible fertilizations in the field, *H* = 0.42 (0.33, 0.53) and *H* = 0.35 (0.30, 0.40), are lower than many estimates previously reported in California *D. simulans*, which ranged from 0.32 to 0.71 [4]. However, the one field estimate from Turelli and Hoffmann [4] derived solely from wild-caught males, the procedure used in this study, produced a very similar result: *H* = 0.43 (0.35, 0.56). In field studies, male age and mating number cannot be controlled and this contributes to variation in *H* estimates. A likely factor contributing to our relatively low estimates is that the flies were collected in a commercial peach orchard, in which the fruit was just ripening, and the *D. simulans* population was expanding. Therefore, the population age structure may have been skewed towards young males, enhancing CI. Our lab studies, discussed below, suggest that recently collected strains of *w*Ri may actually cause less intense CI than found previously.

#### Lab estimates: Uninfected female susceptibility

Our lab studies tested whether either the host or *Wolbachia* had evolved to significantly alter CI intensity. Given imperfect maternal transmission, uninfected females persist in all natural populations; and imperfect transmission quickly breaks down associations between nuclear alleles and infection status [22]. Hence, in polymorphic populations, all *D. simulans* nuclear alleles will find themselves in both infected and uninfected females. Obviously, under these conditions, selection will favor genotypes that suppress CI [11]. Our data (Figures 1 and 2) demonstrate that recently collected females are not able to resist CI induction any more or less than W88 females. Our W88 lab stock has been sheltered from CI for over 20 years, while populations near Davis have been subject to selection to reduce CI since 1990, over 200 generations, assuming that generation times in nature are roughly one month, as estimated in [4].

Our finding that CI-resistance has not evolved in California *D. simulans* is consistent with the data of Hoffmann and Turelli [6] who found no heritable variation in CI-susceptibility among hundreds of isofemale lines collected from Melbourne, Australia in 1985. Jaenike and Dyer [23] present evidence that *D. innubila* has not evolved resistance to *Wolbachia*-induced male killing (MK), despite appreciable selection favoring suppressors for at least 15,000 – and possibly over 500,000 – generations. In contrast, transinfection experiments, introducing the MK *Wolbachia* that naturally infects *D. recens* into its uninfected sister species *D. subquinaria*, reveal that *D. subquinaria* is polymorphic for dominant alleles conferring resistance to MK [24]. Even more striking is the evolution of resistance to MK over the past century in south Pacific and southeast Asian populations of the butterfly *Hypolimnas bolina* [25], [26]. As in *D. subquinaria*, MK resistance in *H. bolina* is dominant [25].

How strong is the selection on *D. simulans* in CA to suppress CI? As shown in [4] and our new data, about 7% of the *D. simulans* females in CA are uninfected and they are likely to lose about half of their potential offspring to CI. Thus, a dominant autosomal suppressor of CI in *D. simulans* would experience a selective advantage on the order of 2%. The fixation probability of a new mutation with this phenotype would be about 4%. Once such an allele reached an appreciable frequency, say about 5%, its near-deterministic trajectory of fixation would take it from 5% to about 50% within 200 generations, the time scale of our field observations in California. These calculations support the arguments of Jaenike and Dyer [23] that dominant suppressors of *Wolbachia*-induced embryo death arise in some taxa extremely rarely or may have strongly deleterious pleiotropic effects that prevent their spread.

#### Lab estimates: CI-induction by infected males

Like Hoffmann and Turelli [6], we found maternally inherited variation in the intensity of CI induced by males from both our D_08_ and Y_09_ collections. The D157 line, which produced no uninfected sublines when initially collected, induced lower CI levels at day 7 than did Riv88. As shown by reciprocal introgressions (Figure 4), the difference in CI intensity was attributed to *Wolbachia* or other maternally inherited factors. Reciprocal crosses also showed that maternal factors caused several Y_09_ lines to produce less intense CI than our older reference stock, Riv84 (Figure 5).

Changes in fecundity have also been associated with evolution of *w*Ri rather than its host [8]. Contrary to the intuition that *Wolbachia* should evolve to increase CI [27], [28], theoretical analyses indicate that *Wolbachia*-produced changes in CI should largely be pleiotropic by-products of changes in fecundity and/or transmission fidelity [11]–[13]. In principle, the generally lower intensity of CI produced by newly collected stocks may be attributable to either upward evolution of *H* in nature or decreases in *H* associated with selection and/or inbreeding in our laboratory stocks. This is currently being investigated.

### Maternal Transmission

Our best estimate of maternal transmission from wild-caught females followed the protocol of the fourth maternal transmission experiment of Turelli and Hoffmann [4]. This involved remating wild-caught females to uninfected males and testing several F2 from each putatively uninfected F1 derived from a wild-caught infected female. Our goal was to estimate in nature *μ*, the fraction of uninfected ova produced by infected females. We first determined *ν*, the fraction of uninfected F2 sublines produced by a wild-caught female. Some uninfected ova from these females may be lost due to CI. This initial experiment produced an estimate *ν* = 0.015, with 95% BC_a_ bootstrap confidence interval (0.003, 0.070), based on bootstrapping over the groups of isofemale sublines produced by individual wild-caught females. After mating these females to uninfected lab males, we obtained *μ* = 0.048 (0.016, 0.116) in our second experiment.

As noted in [4], if we assume that uninfected ova from infected females are as susceptible to CI as are the ova from uninfected females,
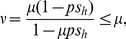
(1)where *p* denotes the infection frequency among mating adults in nature, and *H* = 1−*s_h_* is the relative hatch rate of incompatible crosses in nature. (The numerator in (1) is proportional to the number of uninfected surviving progeny from an infected female, and the denominator is proportional to the total number of surviving progeny for both infected and uninfected females.) Our point estimates from this study are *μ* = 0.048, *p* = 0.932 and *s_h_* = 0.615. Using (1), these produce an estimate of *ν* = 0.021, which is very close to the observed value (0.015) and certainly within the estimated 95% confidence interval (even without accounting for the variation in our individual point estimates).

The new estimate *μ* = 0.048 is essentially identical to the composite estimate *μ* = 0.042 produced in [4], indicating that the mean rate of maternal transmission has not changed substantially in nature. However, several aspects of our data and those in [4] merit comment. First, different experiments in [4] gave very different results. Their first and third maternal transmission experiments estimated *ν* in Eq. (1) from females collected in April 1993 and November 1993 at Ivanhoe in central California. These females were not remated to uninfected males in the lab. In the April sample, of 44 infected wild-caught females that produced 8–10 F2 sublines, 23 produced both infected and uninfected F2 progeny (420 F2 sublines were assayed in total). In the November sample, all 49 infected wild-caught females produced only infected F2 (490 sublines were examined). Using a *χ* ^2^ contingency test, these experiments gave significantly different results concerning the fraction of infected wild-caught females that show imperfect maternal transmission (*P*<10^−7^). Given this extreme temporal variation from a single population, changes in *μ* over decades involving different populations will be essentially impossible to detect.

A striking aspect of our new data is that 4 of 65 wild-caught females accounted for most of the imperfect maternal transmission. Indeed, one female produced only one infected F2 subline out of ten. Our new estimate of *μ* is based on the experimental design used to produce the data in Figure 4 of [4]. In that experiment, 15 of 60 infected females produced both infected and uninfected F2 sublines, and one produced only one infected subline out of 11. Turelli and Hoffmann [4] conjectured that this female may have experienced rare paternal transmission of *Wolbachia*, as described in Hoffmann and Turelli [6]. However, it is equally plausible that females in nature harbor dramatically different levels of *Wolbachia*, which affect maternal transmission rates of *Wolbachia* and susceptibility to CI (see Figure 4 of Turelli and Hoffmann [4]). Unckless et al. [29] documented 20,000-fold variation in *Wolbachia* density among wild-caught *D. innubila*. This variation is associated with both penetrance of the MK phenotype and fidelity of maternal transmission. Similar data are needed for the *D. simulans*-*w*Ri system [4]. In light of plausible extreme variation in *Wolbachia* titer among wild-caught flies, one might question the validity of our PCR assay for infection status, upon which our parameter estimates critically depend. Indirect support for the reliability of our assay comes from a new study by Casper-Lindley et al. [30] that reports a recent survey of *Wolbachia* infection frequencies in central California based on high-resolution microscopic examination of fluorescent-stained ovarioles. In a pooled sample of 106 females collected over three years, they report an infection frequency of 93.4%, consistent with our PCR-based estimate in Table 1.

### Fecundity

Our point estimate of *F*, the relative fecundity of infected females, is 0.98. However, even with over 500 wild-caught females, the fact that only about 7% are uninfected limits the accuracy of this estimate, as reflected by the wide 95% BC_a_ bootstrap confidence interval (0.85, 1.15). Weeks et al. [8] used data from recently collected (2003–2005) versus old (1988) laboratory stocks to argue that *w*Ri had evolved over about 15 years to increase female fecundity. They found that *w*Ri seemed to increase fecundity in the laboratory by about 10%, but argued for much smaller effects in nature, on the order of a few percent. Our new field data are consistent this prediction. Differences in fecundity effects for laboratory versus field populations are analogous to the differences we observe in maternal transmission. In our long-held laboratory stocks, we observe perfect maternal transmission of *w*Ri, whereas we have repeatedly found infected females in nature who produce infected and uninfected progeny [4], [5]. To test the robustness of the Weeks et al. [8] fecundity finding and our new results concerning CI levels, we are now screening large samples of recently collected isofemale lines for variation in both CI and *w*Ri-induced fecundity effects under laboratory conditions.

### Infection Frequency

Sampling over a thousand individuals in 2008, we estimated the *w*Ri infection frequency near Davis, California to be 0.932 (0.915, 0.946). Essentially the same value was obtained by Weeks et al. [8] sampling four populations from southern to northern California in 2003 and by Turelli and Hoffmann [4] from similar sites in late 1993. Is this apparent equilibrium frequency, denoted 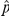, consistent with the simple three-parameter model proposed in Hoffmann et al. [5]? Using our point estimates from the field, *μ* = 0.048, *s_h_* = 0.62 and *s_f_* = 0.0, we obtain 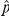 = 0.966, which is significantly higher than the observed value. However, relatively slight changes in the parameter estimates, consistent with their variation over assays, can produce the observed value. For instance, if we set *μ* = 0.05 and *s_f_* = 0.0, we obtain 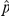 = 0.932 with *s_h_* = 0.47, which is consistent with many of our field estimates, including one of the two reported here. Conversely, if we set *s_h_* = 0.6 and *s_f_* = 0.0, we obtain 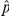 = 0.932 with *μ* = 0.079, which is statistically consistent with our latest estimate, *μ* = 0.048 (0.016, 0.116). Holding *s_h_* = 0.6 and introducing a 5% fecundity cost (*s_f_* = 0.05) reduces the required imperfect transmission rate to *μ* = 0.053. Hence, the simple model with field-estimated parameters is consistent with the observed infection frequencies. However, this test is not particularly stringent. As discussed by Weeks et al. [8] and emphasized by Jaenike [31], the surface of equilibrium infection frequencies as a function of *μ*, *s_h_* and *s_f_* is relatively flat near our field-estimated values.

Despite the apparent quantitative adequacy of our simple model, our data demonstrate that it provides an incomplete description of *w*Ri dynamics in nature. The model ignores age structure, even though the intensity of CI is known to be strongly age dependent [2], [4]. However, given that there are no appreciable effects of *w*Ri on viability, fecundity or development time, an age-structured model with many more parameters is unlikely to help us understand our infection frequency data [32]. Our model also uses a binary description, individuals are either infected or uninfected; whereas our new and old data indicate a continuum of infection states that correspond to different levels of maternal transmission and at least partial incompatibility of some infected females with some *w*Ri males (see Figure 4 of [4]). Only one field test has directly addressed this. Turelli and Hoffmann [4] collected virgin females as they emerged from fruit collected and held in nature, then mated them to wild-caught males from the same population. They found no statistically significant difference in the hatch rate of eggs from *w*Ri-infected females mated with infected or uninfected males. However, the sample sizes were relatively small.

A more elaborate model, with an additional parameter describing partial CI between infected males and infected ova, will clearly lower the predicted equilibrium. In addition to the three parameters *μ*, *s_f_* and *s_h_*, let *H*_1_ = 1−*s_h_*_1_ denote the average relative hatch rate when infected ova are fertilized by sperm from infected males. If we assume that *μ* = 0.05, *s_f_* = 0.0, *s_h_* = 0.5 and *s_h_*_1_ = 0, we obtain 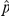 = 0.941 from the standard equilibrium formula (Eq. 3 of [4]). Introducing *s_h_*_1_ = 0.05, i.e., only one-tenth the relative embryo loss attributable to CI between uninfected ova and sperm from infected males, lowers the stable equilibrium to 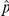 = 0.933.

### Implications and Future Directions

Our laboratory analyses of CI intensity are strongly suggestive of *w*Ri evolution in California over the past 20 years, consistent with the data of Weeks et al. [8]. Future analyses will focus on characterizing many *w*Ri-infected lines for *Wolbachia* load in nature, maternal transmission, fecundity and CI induction, relative to the reference Riv88 and Riv84 stocks. We also hope to associate CI and fecundity phenotypes with *w*Ri genomic variation. The published *w*Ri genome sequence [33] greatly facilitates the assembly of other *w*Ri genomes.

This study produced four primary findings. First, we found relative stability in the parameter values governing the dynamics and equilibria of the Californian *D. simulans*-*w*Ri system after about 15 years and probably around 200 generations. Second, consistent with the results from Hoffmann and Turelli [6], we found no variation among uninfected isofemale lines in their susceptibility to *w*Ri-induced CI. Third, like [6], we found abundant maternally inherited variation in CI induction, presumably associated with variation among the *w*Ri segregating in nature. Fourth, we have tentative evidence that *w*Ri has evolved to produce lower levels of CI. One 2008 isofemale line (D157) has repeatedly shown lower CI intensity than Riv88. Five additional lines from our 2009 collection showed lower CI intensity than Riv84, which in all cases could be attributed to a cytoplasmically inherited factor. Further screening is required to rule out inbreeding artifacts associated with our laboratory stocks, to document the repeatability of the proposed evolutionary changes, and to document the spatial and temporal dynamics of the *w*Ri variants we have described.

## References

[1] Hilgenboecker KP, Hammerstein P, Schlattmann P, Telschow A, Werren JH (2008). How many species are infected with *Wolbachia*? - a statistical analysis of current data.. FEMS Microbiol Lett.

[2] Hoffmann AA, Turelli M, Simmons GM (1986). Unidirectional incompatibility between populations of *Drosophila simulans*.. Evolution.

[3] Binnington KC, Hoffmann AA (1989). *Wolbachia*-like organisms and cytoplasmic incompatibility in *Drosophila simulans*.. J Invertebr Pathol.

[4] Turelli M, Hoffmann AA (1995). Cytoplasmic incompatibility in *Drosophila simulans*: dynamics and parameter estimates from natural populations.. Genetics.

[5] Hoffmann AA, Turelli M, Harshman LG (1990). Factors affecting the distribution of cytoplasmic incompatibility in *Drosophila simulans*.. Genetics.

[6] Hoffmann AA, Turelli M (1988). Unidirectional incompatibility in *Drosophila simulans*: inheritance, geographic variation and fitness effects.. Genetics.

[7] Turelli M, Hoffmann AA (1991). Rapid spread of an inherited incompatibility factor in California *Drosophila*.. Nature.

[8] Weeks AR, Turelli M, Harcombe WR, Reynolds KT, Hoffmann AA (2007). From parasite to mutualist: rapid evolution of *Wolbachia* in natural populations of *Drosophila*.. PLoS Biol.

[9] Hoffmann AA, Turelli M, O'Neill SL, Werren JH, Hoffmann AA (1997). Cytoplasmic incompatibility in insects.. Influential passengers: inherited mircoorganisms and arthropod reproduction.

[10] Werren JH (1997). Biology of *Wolbachia*.. Annu Rev Entomol.

[11] Turelli M (1994). Evolution of incompatibility-inducing microbes and their hosts.. Evolution.

[12] Haygood R, Turelli M (2009). Evolution of incompatibility-inducing microbes in subdivided host populations.. Evolution.

[13] Prout T (1994). Some evolutionary possibilities for a microbe that causes incompatibility in its host.. Evolution.

[14] Poinsot D, Charlat S, Mercot H (2003). On the mechanism of *Wolbachia*-induced cytoplasmic incompatibility: confronting the models with the facts.. Bioessays.

[15] Zhou WG, Rousset F, O'Neill SL (1998). Phylogeny and PCR-based classifications of *Wolbachia* strains using *wsp* gene sequences.. Proc Roy Soc Lond B.

[16] Voelker RA, Gibson W, Graves JP, Sterling JF, Eisenberg MT (1991). The *Drosophila* suppressor of sable gene encodes a polypeptide with regions similar to those of RNA-binding proteins.. Mol Cell Biol.

[17] Karr TL, Yang W, Feder ME (1998). Overcoming cytoplasmic incompatibility in *Drosophila*.. Proc Roy Soc Lond B.

[18] Reynolds KT, Hoffmann AA (2002). Male age, host effects and the weak expression or nonexpression of cytoplasmic incompatibility in *Drosophila* strains infected by maternally transmitted *Wolbachia*.. Genet Res.

[19] Hercus MJ, Hoffmann AA (1999). Dessication resistance in interspecific *Drosophila* crosses: genetic interactions and trait correlations.. Genetics.

[20] Efron B, Tibshirani RJ (1993). An introduction to the bootstrap.

[21] Warren DL, Glor RE, Turelli M (2008). Environmental niche equivalency versus conservatism: quantitative approaches to niche evolution.. Evolution.

[22] Turelli M, Hoffmann AA, McKechnie SW (1992). Dynamics of cytoplasmic incompatibility and mtDNA variation in natural *Drosophila simulans* populations.. Genetics.

[23] Jaenike J, Dyer KA (2008). No resistance to male-killing *Wolbachia* after thousands of years of infection.. J Evol Biol.

[24] Jaenike J (2007). Spontaneous emergence of a new *Wolbachia* phenotype.. Evolution.

[25] Hornett EA, Charlat S, Duplouy AMR, Davies N, Roderick GK (2006). Evolution of male killer suppression in a natural population.. PLoS Biol.

[26] Hornett EA, Charlat S, Wedell N, Jiggins CD, Hurst GDD (2009). Rapidly shifting sex ratio across a species range.. Curr Biol.

[27] Frank SA (1997). Cytoplasmic incompatibility and population structure.. J Theor Biol.

[28] Hurst LD (1991). The evolution of cytoplasmic incompatibility or when spite can be successful.. J Theor Biol.

[29] Unckless R, Boelio LM, Herren JK, Jaenike J (2009). *Wolbachia* as populations within individual insects: Causes and consequences of density variation in natural populations.. Proc Roy Soc Lond B.

[30] Casper-Lindley C, Kimura S, Saxton DS, Essaw Y, Simpson I (2011). A fluorescent-based method for rapid *Wolbachia* detection in the *Drosophila* germline and somatic tissues.. Appl Environ Microbiol.

[31] Jaenike J (2009). Coupled population dynamics of endosymbionts within and between hosts.. Oikos.

[32] Turelli M (2010). Cytoplasmic incompatibility in populations with overlapping generations.. Evolution.

[33] Klasson L, Westberg J, Sapountzis P, Näslund K, Lutnaes Y (2009). The mosaic genome structure of the *Wolbachia w*Ri strain infecting *Drosophila simulans*.. Proc Nat Acad Sci U S A.

