# Correction: A Re-Examination of *Wolbachia*-Induced Cytoplasmic Incompatibility in California *Drosophila simulans*

**DOI:** 10.1371/journal.pone.0138050

**Published:** 2015-09-08

**Authors:** Lauren B. Carrington, Jeremy R. Lipkowitz, Ary A. Hoffmann, Michael Turelli

There is an error in Fig 1: Panel A is a duplicate of Panel B. Please see the correct Fig 1 here.

**Fig 1:**
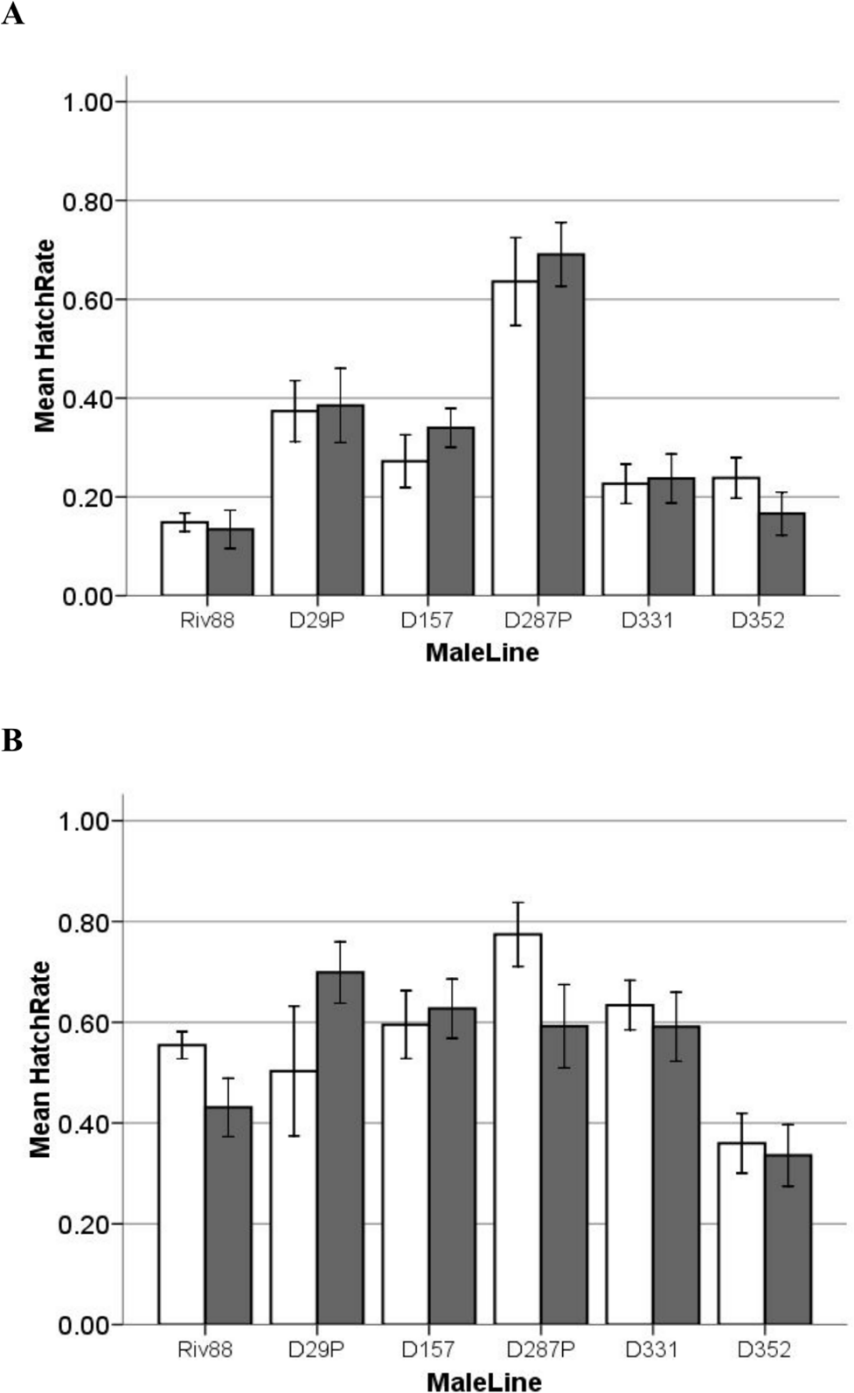
CI induction of old (Riv88) and new (D_08_) males on new (D_08_) and old (W88) uninfected females. Females of newly collected lines are shown in white bars, females from W88 are shown in grey. Error bars show ±1 SE of the mean. Bars indicate the mean hatch of uninfected females when mated to infected males who are: **A**) seven days old, and **B**) fourteen days old.
